# Shining the light on mesenchymal stem cell-derived exosomes in breast cancer

**DOI:** 10.1186/s13287-023-03245-3

**Published:** 2023-02-08

**Authors:** Ghaidaa Raheem Lateef Al-Awsi, Fahad Alsaikhan, Ria Margiana, Irfan Ahmad, Indrajit Patra, Mazin A. A. Najm, Ghulam Yasin, Iroda Rasulova, Ali Thaeer Hammid, Hamzah H. Kzar, Moaed E. Al-Gazally, Homayoon Siahmansouri

**Affiliations:** 1 Department of Radiological Techniques, Al-Mustaqbal University College, Babylon, Iraq; 2 College of Pharmacy, Prince Sattam Bin Abdulaziz University, Alkharj, Kingdom of Saudi Arabia; 3 Department of Anatomy, Faculty of Medicine, Universitas Indonesia, Jakarta, Indonesia; 4 Master’s Programme Biomedical Sciences, Faculty of Medicine, Universitas Indonesia, Jakarta, Indonesia; 5 Andrology Program, Faculty of Medicine, Universitas Airlangga, Surabaya, Indonesia; 6 Dr. Soetomo General Academic Hospital, Surabaya, Indonesia; 7 Department of Clinical Laboratory Sciences, College of Applied Medical Sciences, King Khalid University, Abha, Saudi Arabia; 8 Independent Researcher, Durgapur, West Bengal India; 9 Pharmaceutical Chemistry Department, College of Pharmacy, Al-Ayen University, Thi-Qar, Iraq; 10 Department of Botany, Bahauddin Zakariya University, Multan, Pakistan; 11 Independent Researcher, “Kasmed” Private Medical Centre, Tashkent, Uzbekistan; 12 Computer Engineering Techniques Department, Faculty of Information Technology, Imam Ja’afar Al-Sadiq University, Baghdad, Iraq; 13 Veterinary Medicine College, Al-Qasim Green University, Al-Qasim, Iraq; 14 College of Medicine, University of Al-Ameed, Karbala, Iraq; 15 Department of Immunology, Faculty of Medicine, Tabriz University of Medical Sciences, Tabriz, Iran

**Keywords:** Extracellular vesicles, Tumor, Exosome, Breast cancer, Mesenchymal stem cell

## Abstract

In women, breast cancer (BC) is the second most frequently diagnosed cancer and the leading cause of cancer death. Mesenchymal stem cells (MSCs) are a subgroup of heterogeneous non-hematopoietic fibroblast-like cells that have the ability to differentiate into multiple cell types. Recent studies stated that MSCs can migrate into the tumor sites and exert various effect on tumor growth and development. Multiple researches have demonstrated that MSCs can favor tumor growth, while other groups have indicated that MSCs inhibit tumor development. Emerging evidences showed exosomes (Exo) as a new mechanism of cell communication which are essential for the crosstalk between MSCs and BC cells. MSC-derived Exo (MSCs-Exo) could mimic the numerous effects on the proliferation, metastasis, and drug response through carrying a wide scale of molecules, such as proteins, lipids, messenger RNAs, and microRNAs to BC cells. Consequently, in the present literature, we summarized the biogenesis and cargo of Exo and reviewed the role of MSCs-Exo in development of BC.

## Introduction

Breast cancer (BC) is the second most invasive cancer in the world and the second leading cause of malignancy death among females overall [1, 2]. Several factors including genetic and epigenetic mutations, unusual hormone levels, and environmental agents play an important role in BC development [3]. BC can be classified according to the expression of estrogen receptor-positive, progesterone receptor-positive, and human epidermal growth factor receptor-2-positive [4]. Also, triple-negative breast cancer (TNBC) is a class of aggressive BC lacking the expression of ER, PR, and HER2 [5].

Mesenchymal stem cells (MSCs), also mentioned as mesenchymal stromal cells, are non-hematopoietic multipotent cells, chiefly found in the bone marrow (BM) that possess the ability of self-renewal and also display multilineage differentiation [6–8]. MSCs were identified in variety of adult and fetal/perinatal tissues, such as BM, placenta, Wharton’s jelly, adipose tissue (AD), human umbilical cord (hUC), peripheral blood, and dental pulp [9–12]. MSCs can display diverse features according to their origin; however, they must show three minimal principles defined by the International Society for Cellular Therapy [13]. First, MSCs have plastic-adherence capability when maintained in growth culture media. Second, MSCs must have a particular cell surface antigen expression such as CD73, CD90, CD105, and lacking expression of CD45, CD34, CD14 or CD11b, CD79α or CD19 and HLA-DR. Third, these cells should have the capacity to differentiate into various mesodermal cell types (i.e., adipocytes, chondrocytes, and osteoblasts) when cultured under special conditions. Furthermore, MSCs have ability to differentiate into non-mesodermal cells, such as neuronal cells, cardiomyocytes, hepatocytes or epithelial cells [14–17]. This property of stromal cells provides advantages in tissue regeneration. MSCs own a homing ability, which can recruit into the inflammation sites and tissue repair [18–21]. Moreover, MSCs have various biological roles such as multilineage differentiation, immunosuppression, and tissue-repair development [22, 23]. Because of these benefits, MSCs have been broadly used in clinical studies [24–28], including spinal cord injuries, chronic obstructive pulmonary disease, renal failure, Parkinson’s disease, COVID19, and autoimmune diseases (https://clinicaltrials.gov/).

Multiples studies have been reported that MSCs can also transfer to the tumor stroma and contribute to tumor microenvironment formation [29–31]. The results of studies have demonstrated that MSCs can change the tumor microenvironment depending on the requirements of the cancer cells for tumor growth directly by releasing growth factors or increasing tumor angiogenesis [32–34]. On the other hand, some studies indicated that MSCs may contribute in suppressing tumor cells [35–37]. Nevertheless, the precise mechanisms underlying these opposite effects remain unclear. A large number of studies have been conducted to investigate MSC-derived exosomes (MSCs-Exo) and revealed that MSCs-Exo have mechanisms similar to those of MSCs, such as regeneration tissue injury, repressing inflammatory responses, tumor progress, and stimulating angiogenesis [38–42].

In addition, MSCs-Exo involve in the influences of MSCs on tumor progress. Multiple investigations showing the effects of MSCs-Exo on tumor progress. Therefore, it is reasonable to hypothesize that MSCs-Exo transfer crucial MSCs-related molecules that alter the physiology of target cells in a particular manner. In recent years, MSCs-Exo have shown emerging role in cell-to-cell communication in the promotion of malignancies and cancers.

In this review, first we will describe the exosome’s biogenesis and component. Then we will highlight the effects of MSCs-Exo on breast cancer cell growth and progression.

### Extracellular vesicles overview

In addition to the secretion of secretory vesicles by specific cells that facilitates the vesicular transport of cargos, such as hormones or neurotransmitters, most of cells are able to release several types of membrane vesicles, known as extracellular vesicles (EVs) [43]. At the beginning, it was thought that the release of EVs was a means of eliminating unnecessary compounds from the cell [44]. Nowadays, we know that EVs are more than just waste transporters, and the chief attention in this field is now focused on their capability to transport cargos between cells, such as DNA to lipids and proteins. EVs are important signaling vehicles in cell homeostatic processes or in pathological progression. Based on their origin, EVs can be mostly divided into two important classes: Exo and microvesicles (MVs) [10, 45]. The term Exo was firstly used for membrane vesicles ranging from 30 to 100 nm in diameter secreted by reticulocytes during RBC maturation [44, 46, 47]. Basically, Exo is intraluminal vesicle (ILV) generated via the internal budding of endosome during development of multivesicular bodies (MVBs), which are intermediated into endosomal process, and released upon merging with the plasma membrane (PM) [48, 49]. In the 1990s, Exo were stated to be released by immune cells with potential roles associated with immune regulation and were suggested for apply as vehicles in anti-tumoral immune responses [50, 51]. The results of studies have demonstrated that various different cell types secrete Exo, and their effects in cell-to-cell communication in wide range of information related to normal and pathological conditions are now well documented [52, 53].

**Fig. 1:**
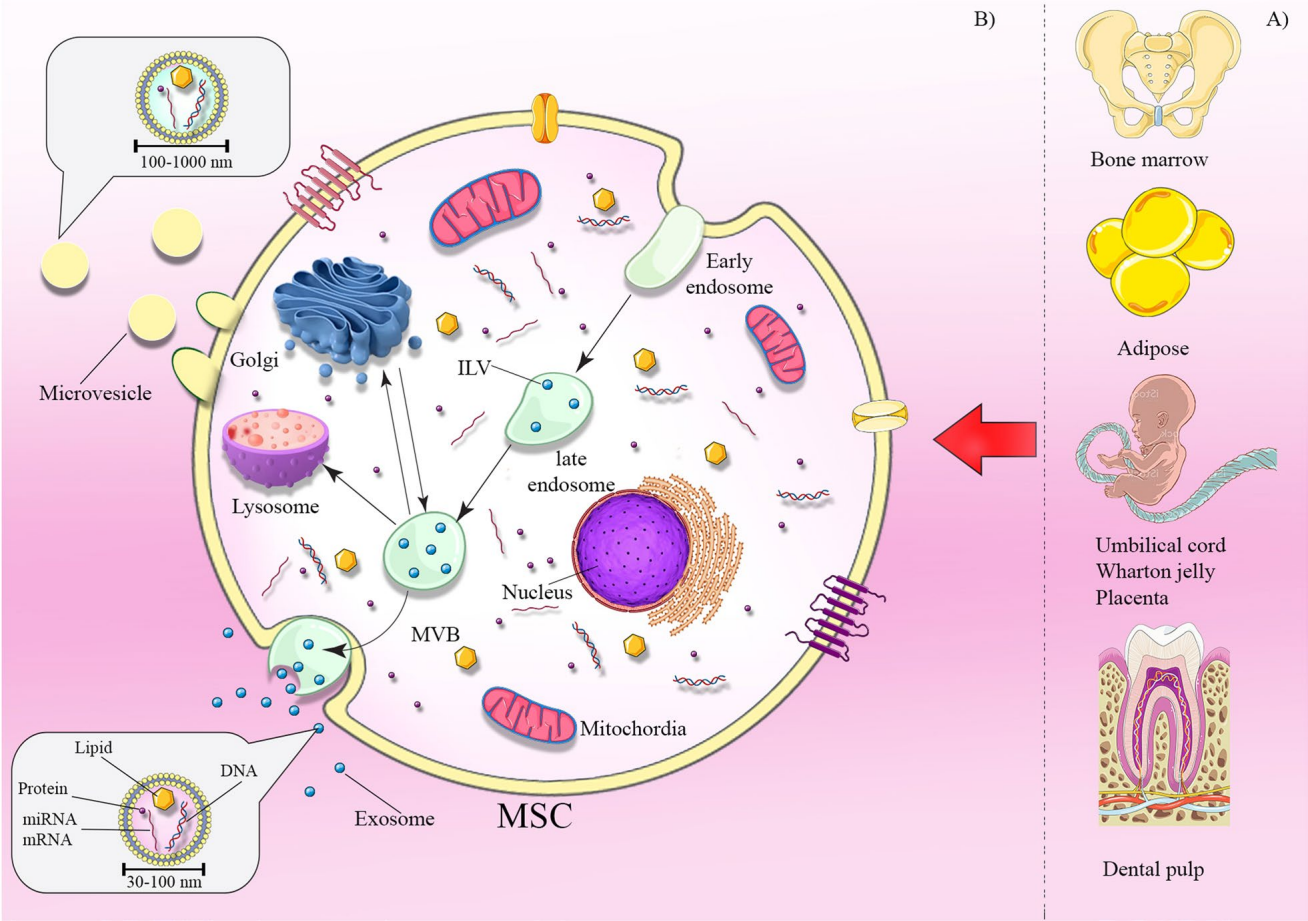
Sources of MSCs and exosome biogenesis. MSCs can be obtained from various sources including bone marrow, adipose, umbilical cord, Wharton jelly, placenta, and dental pulp (**a**). Mechanisms of exosome biogenesis and secretion (**b**). Exosome biogenesis initiates with the process of endocytosis. It includes internal budding of the cell membrane and embraces bioactive molecules, resulting to the formation of the endosome. Furthermore, these molecules are sorted in smaller vesicles which bud from membrane into endosome lumen forming multivesicular bodies (MVBs). The MVBs either fuse with lysosome for degradation or fuse with the plasma membrane to release exosomes

In the beginning, MVs termed ‘platelet dust’, were initially stated as subcellular ingredients originating from thrombocytes in plasma and serum of healthy populations [54]. Subsequently, stimulated neutrophils displayed a process that released the vesicles via fusion with PM, called exocytosis [55]. In spite of the fact that MVs have been studied mostly for their function in blood coagulation, they participate in intracellular communication in many cell types, such as tumor cells, where they are mainly termed oncosomes [56, 57]. MVs are generally larger than Exo (100–1000 nm in diameter) and generated by direct budding of the PM. This process is mediated via an elevation of intracellular cytosolic calcium that triggers calpain. Consequently, this cause restoration of the cytoskeleton, by cleaving the actin protein network and finally budding occur Fig. 1 [58–61].

### Exosomes biogenesis

Exo generate with internal budding of the PM to produce early endosome, in which early endosomes membrane invaginate and envelope surrounding lumina with cytoplasmic content cause the formation of ILVs within large MVBs [62, 63]. Finally, MVBs get transferred to PM through cytoskeletal and microtubule network and fusion with the PM that the ILVs get released as Exo. In addition, MVBs have another fates, they were delivered to lysosomes for degradation of their components, or transported to the trans-Golgi system for endosome recycling [64]. The mechanisms that regulate the fate of MVBs are not absolutely clear. Two members of the Rab family, Rab27A and B, stimulate MVBs transportation to cell border then SNARE complex induces MVBs fusion with the cell membrane to secrete Exo [65]. Evidence has shown that endosomal-sorting complex required for transport (ESCRT) plays a central function in ILVs formation [66]. This complicated machinery composed of multiprotein complexes (ESCRT-0, I, II, and III) with associated proteins (Tsg101, ALIX, and VPS4) that work cooperatively to promote generation of MVB, vesicle formation, and protein cargo sorting [67, 68]. During the biogenesis process, each complex has the function, ESCRT-0 recognizes and sequestrates the ubiquitinated cargos to specific domains of the endosomal membrane and trigger the pathway. ESCRT-I and II complexes initiate the deformation of membrane causing buds or stable membrane, the total complex will then merge with ESCRT-III, a subset of ESCRT that is implicate in stimulating the budding processes. Eventually, after cleaving the buds to generate ILVs, Vps4 is recruited to ESCRT-III in order to separate it from the cytoplasmic membrane [69–71]. Furthermore, exosomal protein Alix, that is related to some ESCRT (TSG101 and CHMP4) proteins, contributes to endosomal membrane budding, as well as exosomal components selection through interaction with syndecan [72].

Interestingly, last evidence showed an another route for Exo biogenesis and their cargo sorting into MVBs in an ESCRT-independent pathway, which contains lipids and related protein as tetraspanins [73].

### Exosomes components

**Fig. 2:**
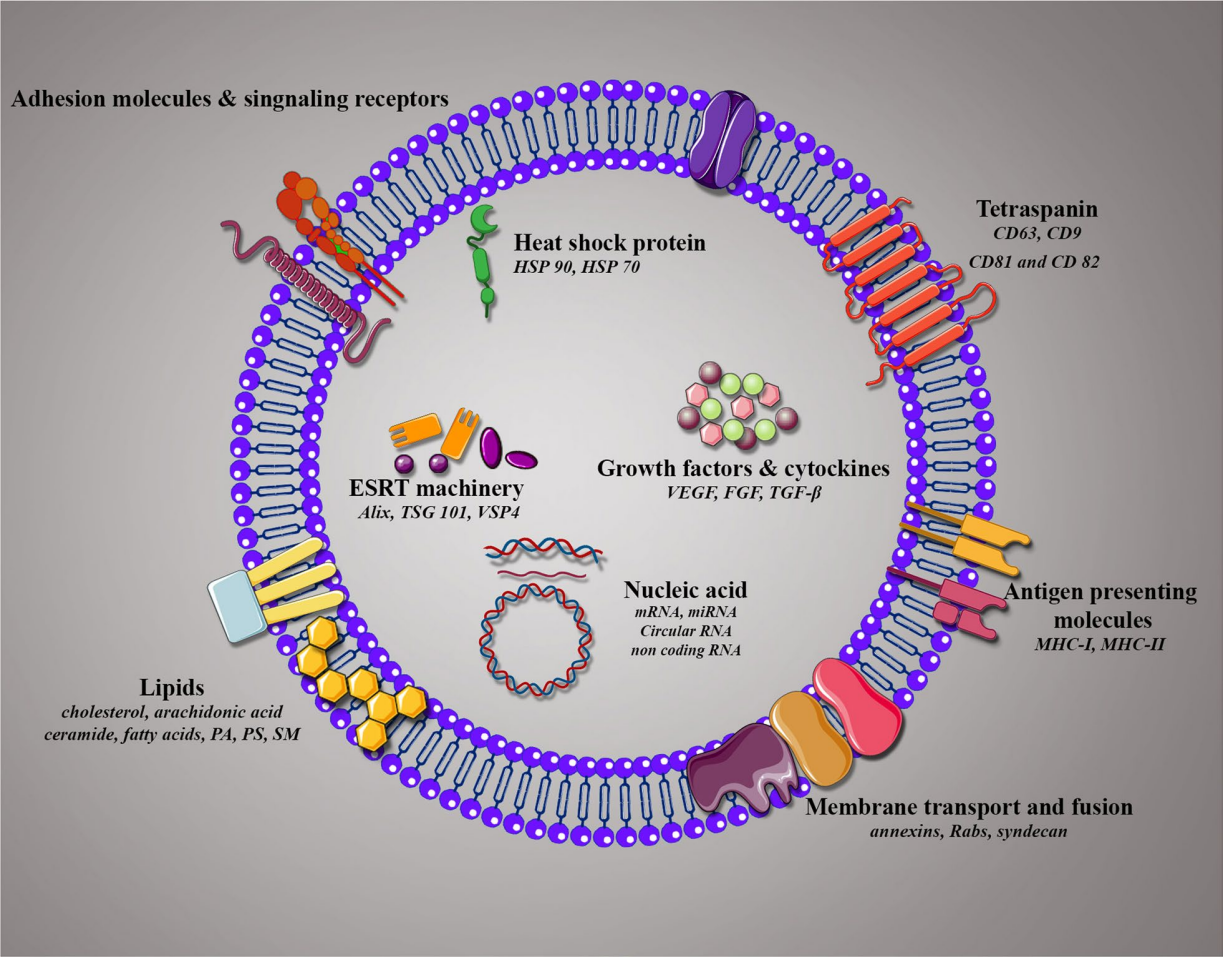
Composition and membrane orientation of exosomes. Schematic illustration of exosome composition containing several families of proteins, lipids, and nucleic acids with membrane orientation in exosomes

Exo typically contain luminal components, such as nucleic acids (DNA, RNA), lipid structures, proteins, peptides, and amino acids enriched in a lipid bilayer membrane (Fig. 2). Classically, Exo are highly enclosed in proteins with several roles, including tetraspanins (CD9, CD63, CD81, and CD82), which mediate cellular penetration, invasion, and fusion events, heat shock proteins (HSP70, HSP90), which participate in antigen processing and binding; MVB generation proteins that take part in Exo release (Alix, TSG101); as well as proteins responsible for membrane carrying and fusion (annexins and Rab) [74–76]. Exo also comprise of various forms of RNAs that can be incorporated into target cells. According to the evidences, microRNAs (miRNAs) are the most plentiful in exosomal RNA classes. There are other types of RNAs, such as ribosomal RNA, long non-coding RNA, transfer RNA, messenger RNA (mRNA), and small nuclear RNA [77]. When miRNAs transferred to Exo, they can experience unidirectional transport between cells, leading to the creation of a cell-to-cell trafficking network, which, in turn, causes transient or constant phenotypic modifications of target cells [78]. The miRNAs, such as miR-1, miR-320, miR-15, miR-214, miR-29a, miR-16, miR-151, miR-375, and lethal-7, have critical roles in angiogenesis, hematopoiesis, exocytosis, and tumorigenesis [79–81]. Intriguingly, evidence has been revealed that long RNA types, specifically long non-coding RNAs (lncRNAs) and circular RNAs (circRNAs) are expressed in Exo, and impact a range of biological processes such as tumor progression [82]. Several studies have been reported that expression of lncRNAs including lncRNA BCRT1, lncRNA HOXD-AS1, lncRNA UCA1, and lncRNA LNMAT2 were considerably increased in Exo derived from tumor cells, which was functionally implicated in promotion cancer cell growth, metastasis, and angiogenesis [83–86]. These studies revealed that Exo can mediate transfer of lncRNA as a significant mechanism in tumor progression and play an important role in modification of the tumor microenvironment through influencing main cellular pathways. The biological function of Exo base on not only in their proteins and nucleic acids, but in their lipid composition. Commonly, Exo are rich in phosphatidylserine (PS), phosphatidic acid (PA), cholesterol, sphingomyelin (SM), arachidonic acid and other fatty acids, prostaglandins, and leukotrienes, ensure their stability and rigidity [87].

### The roles of MSCs-Exo in tumors

The tumor microenvironment (TME) is a complicated and heterogeneous network incorporating both endothelial cells and immune cells, such as myeloid-derived suppressor cells (MDSCs), tumor-related stromal cells, cancer-associated fibroblasts (CAFs), and tumor-associated macrophages (TAMs). Generally, the TME is crucial for growth and progression of tumor cells.

The role of MSCs-Exo in development of tumor has been shown. According to the growing evidences, MSCs-Exo transport regulatory cargos to a variety of target cells to change the function of TME, such as tumor cell, CAFs, TAMs, MDSCs, and endothelial cells [88]. Studies have exhibited that MSCs-Exo can inhibit [42] and promote [89] tumor development in recipient cells by transferring components in diverse situations and various stages of cancer. Interestingly, MSCs-Exo obtained from diverse tissues evoke difference effects on cancers. For example, bone marrow MSCs-Exo (BM-MSCs-Exo) stimulate the development of cancer cell by transferring different microRNAs [90–92]. However, hUCMSC-derived Exo transferred miR-503-3p down-regulated MEST to inhibit human endometrial cancer cells progression [93], and ADMSC-derived Exo stimulate the differentiation of Th17 and T reg from naive CD4 + T cells to retard proliferation ability of tumor cells via transferring miR-10a [94]. Furthermore, approximately all researches on TA-MSCs-Exo proposed that they can elevate tumor progression [95]. The following parts focus on the capacity of MSCs-Exo in the pathogenesis of breast cancer.

### The roles of MSCs-Exo in breast cancer

**Fig. 3:**
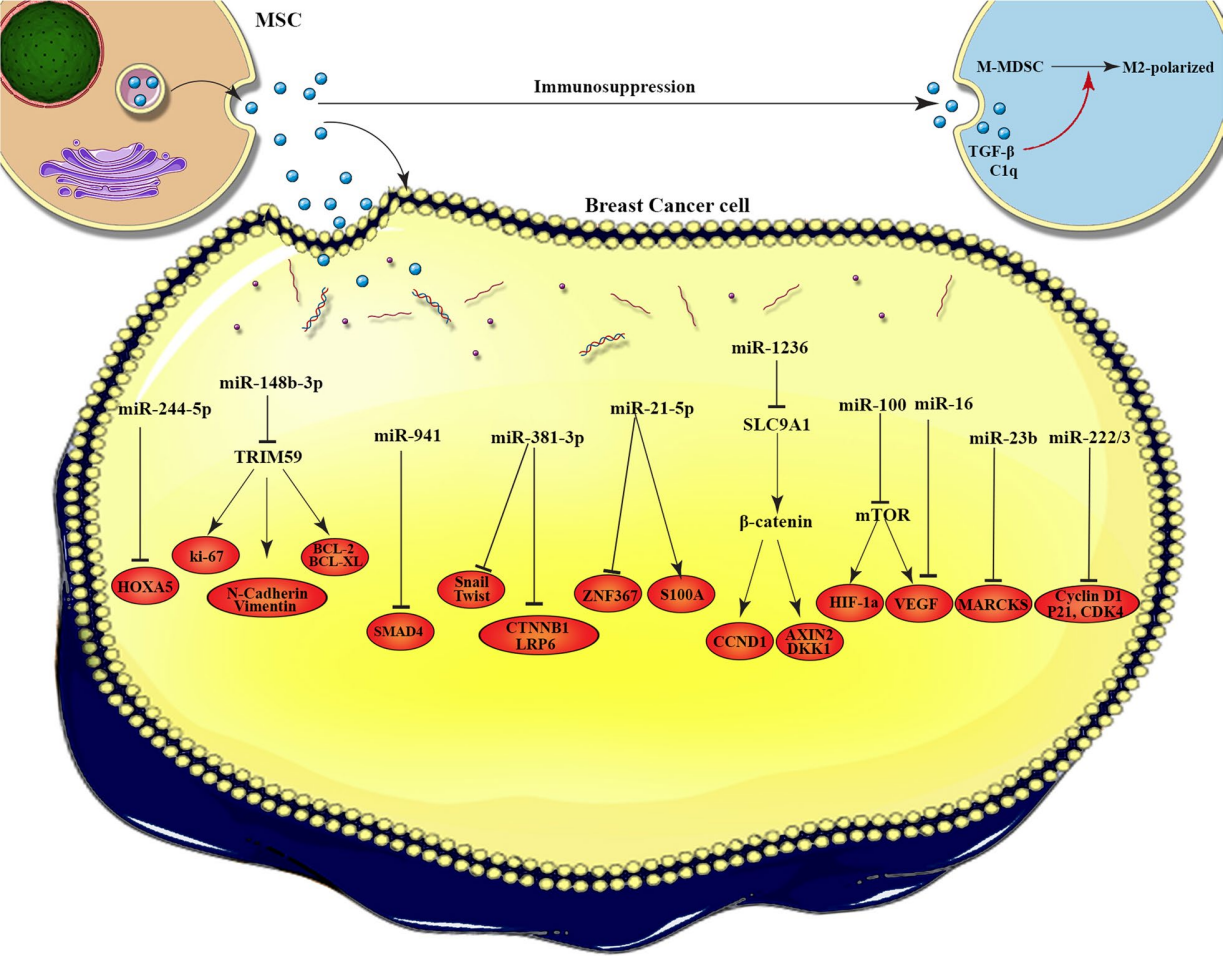
The molecular mechanisms of MSC-EVs cargos in breast cancer progression. This figure illustrates how the exosomes interact with the target cells and the molecular mechanisms by which cargos loaded in exosomes affect breast cancer cells. miR-224-5p increase autophagy in BC cells by down-regulation of HOXA5. miR-148b-3p promotes cell apoptosis via targeting TRIM59. miR-941 suppresses EMT and migration of BC cells by up-regulation of E-cadherin and down-regulation of vimentin, SMAD4, and SNAIL. miR-381 reduces migration, and invasion of BC cells by targeting Wnt signaling pathway and EMT transcription factors. miR-21-5p improves cell viability and drug resistance in the BC cells through induction of S100A6, moreover suppress the metastasis of tumor cell by targeting ZNF367. In addition, miR-1236 increases the sensitivity of BC cells to chemotherapy agent by silencing of SLC9A1 and inactivation of Wnt/β-catenin. Angiogenesis was suppressed in BC cells through miR-100 that modulate the mTOR/HIF-1α axis and miR-16 that down-regulate the expression of VEGF. BC dormancy was promoted by miR-23b that inhibit MARCKS. Quiescence and drug-resistance in tumor cell were induced by miR-222/223 that down-regulate the level of CDK4, Cyclin D1 and p21WAF1

According to the literature, MSCs-Exo can affect tumor development via various mechanisms (Fig. 3, Table 1).

**Table 1:** The role of MSC-Exo in breast cancer tumorigenesis

Tumor progression approach	Origin of Exo	Setting	Cargo	Mechanism	Outcome	References
Angiogenesis	BMSCs	In vitro	NS	NS	Pro-angiogenesis	[101]
		In vivo				
	BMSCs	In vitro	miR-16	Down-regulation of VEGF	Anti-angiogenesis	[102]
		In vivo				
	BMSCs	In vitro	miR-100	Down-regulation of VEGF by modulating the mTOR/HIF-1α signaling axis	Anti-angiogenesis	[107]
Cell proliferation, apoptosis and autophagy	BMSCs	In vitro	miRNAs-21/34a	NS	Induction of cell proliferation	[101]
		In vivo			Reduction of apoptotic	
	BMSCs	In vitro	miR-106a-5p	NS	Induction of cell proliferation	[114]
		In vivo				
	AD-MSCs	In vitro	NS	Activation of two key downstream effector proteins of Hippo, the YAP and TAZ	Induction of cell proliferation	[109]
		In vivo			Reduction of apoptotic	
	AD-MSCs	In vitro	NS	Activation of Wnt/b-catenin signaling pathway by targeting Axin2 and Dkk1	Induction of cell proliferation	[111]
	AD-MSCs	In vitro	miR-145	Modulating of ROCK1, MMP9, ERBB2, and TP53	Suppression of cell proliferation	[117]
					Induction of apoptosis	
	hUCMSCs	In vivo	miR-224-5p	Down-regulation of HOXA5	Induction of cell proliferation	[123]
					Induction of autophagy	
					Reduction of apoptotic	
	hUCMSCs	In vitro	miR-148b-3p	Down-regulating of TRIM59 lead to inhibition of Ki-67, Bcl-xl, Bcl-2, N-cadherin and vimentin expression	Suppression of cell proliferation	[124]
		In vivo			Induction of apoptosis	
	DPSCs	In vitro	miR-34a	NS	Suppression of cell proliferation	[116]
Metastasis	BMSCs	In vitro	miR-106a-5p	NS	Induction of migration and invasion	[114]
		In vivo				
	BMSCs	In vitro	miR-let-7f	NS	Suppression of migration	[130]
	AD-MSCs	In vitro	NS	Activation of the Wnt signaling pathway	Induction of metastasis	[111]
	AD-MSCs	In vitro	miR-145	Suppression of MUC1	Suppression of metastasis	[117]
	AD-MSCs	In vitro	miR-381	Down-regulation of Wnt signaling pathway genes (LRP6 and CTNNB1) and EMT transcription factors (Twist and Snail)	Suppression of migration and invasion	[129]
	hUCMSCs	In vitro	miR-148b-3p	Down-regulation of TRIM59	Suppression of EMT and migration	[115]
		In vivo				
	hUCMSCs	In vitro	miR-21-5p	Down-regulating of ZNF367	Suppression of metastasis	[128]
Dormancy	MSCs	In vitro	NS	NS	Induction of dormancy	[134]
	MSCs	In vitro	NS	Drive BC cell into CSCs through regulation of Wnt/β-catenin pathway	Induction of dormancy	[135]
	BMSCs	In vitro	miR-222/223	Induction of BC cells cycling quiescence by reduction of CDK4, Cyclin D1 and p21WAF1 expression	Induction of dormancy	[136]
		In vivo				
	BMSCs	In vitro	miR-23b	Suppression of MARCKS	Induction of dormancy	[138]
		In vivo				
	AD-MSCs	In vitro	miR-941	Up-regulation of E-cadherin and down-regulation of vimentin, SMAD4, and SNAIL	Induction of dormancy	[137]
Chemotherapy resistance	BMSCs	In vitro	miR-21-5	Upregulation of S100A6	Induction of chemoresistance	[144]
		In vivo				
	AD-MSCs	In vitro	miR-1236	Down-regulation of SLC9A1 and inactivation of Wnt/β-catenin	Induction of chemoresistance	[145]
Immune evasion	MSCs	In vitro	TGF-b, C1q, and semaphorins	Up-regulation of PD-L1 and elevate L-Arginase and IL-10 to stimulate M-MDSC differentiation into M2-polarized macrophages for immunosuppressive	Immunosuppression	[151]
		In vivo				

#### Angiogenesis

Angiogenesis is the generation of new capillaries from pre-existing blood vessels that supplies nutrients for tumor cells, therefore has a crucial role in the BC progression [96]. Proliferation and metastasis of tumor cells based on a sufficient supply of oxygen and nutrients. Accumulated evidence has shown that MSCs-Exo contain angiogenic stimulatory factors such as fibroblast growth factor (FGF), vascular endothelial growth factor (VEGF), TGFβ1, and interleukin-8 (IL-8), which can promote angiogenesis [97, 98]. VEGF acts as a major mediator of angiogenesis in malignancy, it is elevated by hypoxia, wide scale of growth factors, and oncogene expression, and can stimulate mitosis, cell migration, and elevate vascular permeability [99, 100]. It has been documented that BC cells co-infused with BMSCs-Exo have higher angiogenesis capability [101]. However, according to the literature, MSCs-Exo can regulate BC angiogenesis by the targeting effects of some miRNAs on VEGF in BC. Lee and colleagues have reported that BMSCs-Exo contained miR-16 which reduce the expression of VEGF in 4T1 cells and thus suppress angiogenesis and tumor progression in vitro and in vivo [102]. Additionally, the transcription factor hypoxia-inducible factor-1alpha (HIF-1α) regulates VEGF via binding to the hypoxia response element within the VEGF gene promoter [103]. Rapamycin (mTOR) plays an important role in regulating cell proliferation and angiogenesis of endothelial cells [104, 105], exerts a key function in the HIF-1α-mediated expression of VEGF in BC cells [106]. Pakravan et al. have been shown that BMSCs-Exo carrying miR-100 down-regulated the expression of VEGF by modulating the mTOR/HIF-1α axis, consequently suppressed angiogenesis in BC cells. In addition, they represented BMSCs-Exo transferring miR-100 decreased VEGF in a dose-dependent and time-dependent [107]. In summary, although some of studies have confirmed that MSCs-Exo have a promoting effect on BC angiogenesis, most of evidence have suggested their inhibitory effect.

#### Cell proliferation, apoptosis and autophagy

The role of MSCs-Exo in proliferation of cancer cells has been reported. Various studies have stated that MSCs-Exo can exert different effects on the growth of cancer cells [108]. Vallabhaneni and coworkers have been shown that BMSCs-Exo increase survival and proliferation of MCF7 cells through carrying tumor supportive proteins, miRNAs, lipids and metabolites [101]. Similarly, Wang et al. demonstrated that AD-MSCs-Exo promote proliferation and migration of BC cells in vitro and in vivo. They have also found the Hippo signaling pathway was responsible for the tumor progression effects of AD-MSCs-Exo, in which Exo exert their effects through activation of two key downstream effector proteins of Hippo, the YAP and TAZ. Hippo signaling pathway plays a key role in controlling tumorigenesis and regulating BC proliferation and metastasis [109]. However, their study has a limitation, the in vivo model was carried out using adipocytes treated with GW4869 whereas in vitro studies were done with pure Exo.

Furthermore, Wnt/β-catenin signaling involve in various cancer development, which is marked by nuclear accumulation of β-catenin [110]. It has been exhibited that AD-MSCs-Exo can enhance the proliferation of MCF7 cells, and up-regulate the expression of β-catenin and Wnt target genes, such as Axin2 and Dkk1 [111]. These observations suggested that AD-MSCs-Exo play a main role in BC cell proliferation via activation of Wnt signaling pathway. Recently, several studies have been indicated that HAND2-AS1 have an inhibitory effect on BC cell proliferation [112, 113]. In a study conducted by Li. Xing et al. [114], in TUBC, the releasing of BMSCs-Exo carrying miR-106a-5p was inhibited by lncRNA HAND2-AS1. They also reported that HAND2-AS1 can perform as a sponge of miR-106a-5p, and HAND2-AS1 was reduced in TNBC cells. Moreover, up-regulation of HAND2-AS1 suppressed the secretion of BMSCs-Exo comprising miR-106a-5p, thereby inhibited TNBC growth both in vitro and in vivo [114]. Thus, we hypothesize that BMSCs-Exo carrying miR-106a-5p may support TNBC progression. However, a recent study by Yuan et al. demonstrated that exosomal miR-148b-3p from hUCMSCs suppressed proliferation of MDA-MB-231 cell by down-regulating of TRIM59 [115]. Likewise, dental pulp-derived MSCs-Exo with overexpressed miR-34a exerted a suppressive effect on BC cell proliferation [116]. A study conducted by Sheykhhasan et al. [117] has reported that AD-MSCs-Exo containing miR-145 can suppress the proliferation of BC cells through modulating ROCK1, MMP9, ERBB2, and TP53 gene expression [117].

Apoptosis is a process of programmed cell death that occurs to maintain physiologic tissue homeostasis. Apoptosis is activated when a cell is no longer needed or has sustained severe injury [118, 119]. Autophagy is a self-degradative mechanism, in which cytoplasmic proteins or organelles encapsulated in double-membrane vesicles and fused with lysosomes for degradation or renewal [120]. Accumulating evidence exhibited that autophagy plays a key role in the removal of injured or unwanted proteins and cellular organelles, therefore preventing apoptosis and improving survival [121]. It is thought that autophagy inhibits tumor progression. Contrarily, when the tumor is established, autophagy can promote the tumor cell survival and growth [122]. An in vivo study conducted by Wang et al. [123] has been demonstrated that hUCMSCs-Exo carrying miR-224-5p can increase autophagy in BC cells through down-regulation of HOXA5, thereby elevate the proliferation and suppress apoptosis of BC cells. Nevertheless, MSCs-Exo can also exhibit an anti-tumor activity through suppression of apoptosis. Yuan et al. have been represented that hUCMSC-Exo carrying miR-148b-3p exhibited the ability to promote the MDA-MB-231 cell apoptosis via targeting tripartite motif 59 (TRIM59) [115]. The results of studies showed that TRIM59 regulates BC cell apoptosis [124].

In sum, MSCs-Exo can abnormally activate the signaling pathways or carry tumor supporters to increase proliferation of BC cells. However, they can transport tumor suppressors to exert an inhibitory effect on BC cell proliferation. In addition, MSCs-Exo exerted dual effects on autophagy and apoptosis of BC cells that is needed to be further investigate.

#### Metastasis

BC metastasis is one of the chief reasons for the high mortality rate of BC. Diagnosis of BC metastasis in the early stage is crucial for the administration and prediction of BC progression. Metastasis is a process that allows cancer cells to circulate in the bloodstream and lymphatic vessels, and then spread to distant parts of the body for colonization [125, 126]. Recent evidence suggested that epithelial-to-mesenchymal transition (EMT) could increase metastasis of neighboring tumor cells [127]. The results of studies indicated that a complex set of signaling pathways regulate the metastasis [5]. In support of this view, some studies have showed that AD-MSCs-Exo stimulated BC cells migration through the activation of the Wnt signaling pathway [111]. Besides, Exo can promote metastasis through the regulation of other factors. According to the literature, MSC-exosomal-miR-106a-5p elevated both invasion and migration potential of TNBC cells, nonetheless lncRNA HAND2-AS1 can suppress this effect [114].

On the other hand, MSCs-Exo also have the capacity to inhibit the migration and metastasis of BC cells. For example, metastasis gene mucin 1 (MUC1), as a marker of tumorigenesis, overexpressed in BC cells and is related to metastasis. Meanwhile, it can be suppressed as a target of AD-MSCs-Exo containing miR-145 to inhibit the metastasis of T-47D cells [117]. Similarly, it has been revealed that hUCMSCs-Exo-carrying miR-148b-3p inhibit EMT and migration of MDA-MB-231 cells by downregulating the expression of TRIM59 both in vivo and in vitro [115]. In another study, it was found out that husMSC-Exo suppressed the metastasis of MCF-7 cells through delivering miR-21-5p and suppressing ZNF367 expression [128]. One study revealed that miR-381 loaded AD-MSC-Exo reduces migration, and invasion of TNBC cells by targeting Wnt signaling pathway (down-regulation of LRP6 and CTNNB1) and EMT transcription factors (Twist and Snail) [129]. Considerably, BMSCs-Exo with overexpressed miR-let-7f can reduce the proliferation and migration of 4T1 BC cells [130].

In conclusion, MSCs-Exo exert the dual role in BC cell migration and metastasis by regulation of signaling pathways such as wnt/ β-catenin, and miR‑21‑5p/ZNF367 pathway and delivering their cargos such as miRNAs to BC cells.

#### Dormancy

Tumor dormancy is a process that cells experience an arrest of cell cycle, termed quiescence. Dormancy is vital for tumor cells to obtain further mutations, to survive in a new environment, initiate metastasis, and to escape the immune system [131]. There are two types of tumor dormancy; cellular is characterized by three properties: (i) minimum proliferation; (ii) minimum death; and (iii) reversibility that referring to a reversible, non-proliferative, but viable cell status. Another type is tumor mass dormancy that is a situation in which there is a balance between the increase in tumor cell and the decrease in cell death [132, 133]. Casson et al. have been reported that BM-MSCs-Exo initiate an epithelial cell phenotype with decrease proliferation and increase adhesion of MCF7 cells, that suggested dormancy of BC cells [134]. The drawback of this study was that the isolated exosomes contain larger microvesicles that is need to assess the effect of pure MSCs-Exo on BC dormancy. Another study revealed that BM-MSCs-Exo induce BC cell dormancy as cancer stem cells (CSCs). Once BC cells migrate to BM niche and encounter by MSCs, the cargo of BM-MSCs-Exo, alter the behavior of BC cells as CSCs, directly and indirectly by Exo secretion through Wnt/β-catenin pathway [135]. In addition, dormant BC cells can stimulate more cancer cells to enter a dormant state through stimulating MSCs to secret Exo-containing miRNAs. The results of investigations illuminated that miR-222/223 were up-regulated in Exo from BC-primed MSCs, and down-regulated the level of CDK4, Cyclin D1 and p21WAF1 to induce quiescence and drug-resistance within tumor cells [136]. In another study, it was exhibited that AD-MSCs-Exo carrying miR-941 significantly up-regulated the expression of E-cadherin and down-regulated vimentin, SMAD4, and SNAIL to suppress EMT and migration of BC cells following co-culture with MCF7-luminal and MDA-basal cells subtypes, which arrest the cell cycle into dormancy [137]. Furthermore, Ono et al. [138] reported that some miRNAs in BM-MSCs-Exo were enhanced following co-culture with BM-metastatic human BC cell line (BM2). The overexpression of miR-23b promoted BC dormancy by targeting MARCKS that encodes a protein that increase cell cycling and migration. Thus, we postulated that exosomal miR-23b and its inhibition of MARCKS is one of the ways participating in cell inhibition and dormancy in BCSCs. However, they reported that while Exo-treated BM2 cells showed significantly reduced tumor proliferation, BM2 cell overexpressing miR-23b did not show the same grade of growth suppression, recommending that other factors also participate in this effect. According to all studies above, MSCs-Exo own a promoting role in dormancy of BC.

#### Chemotherapy resistance

Chemotherapy is one of the main treatment approaches for cancer that have spread from the primary tumor site. Chemotherapy resistance is an important hindrance to achieve therapies in patients and is the crucial cause of death in most progressive stage cancers [139, 140]. Drug resistance caused by several factors, including genetic mutations and/or epigenetic changes, and other cellular and molecular mechanisms [141]. Growing evidence has demonstrated that MSCs contribute to resistance of cancer cells to wide range of anti-cancer drugs [142]. Some studies have reported that hUCMSCs-Exo can participate in drug resistance of cancer via activation of various signaling cascade, the increase of multi-drug resistance-related proteins, and the inhibition of chemotherapy-induced apoptosis [143]. Several studies suggested that MSCs-Exo can affect chemotherapy agents in BC. Studies conducted in the last part of the last decade indicated that BM-MSCs-Exo promote resistance of BC cells to doxorubicin and the Exo released by doxorubicin-treated MSCs can give rise to a higher resistance of BC cells to doxorubicin than BM-MSCs-Exo. They also have found that doxorubicin treatment enhanced the expression of miR-21-5p in MSCs-Exo, causing the induction of S100A6 in the BC cells both in vitro and in vivo, and improving cell viability and drug resistance [144]. On the other hand, in a study conducted by Jia et al. AD-MSCs-Exo have shown that transferring miR-1236 increased the sensitivity of BC cells to cisplatin through down-regulation of SLC9A1 and inactivation of Wnt/β-catenin [145].

Briefly, these observations propose that MSCs-Exo play a contradictory approach in mediating chemoresistance in BC. More investigation is required to find out the role of MSCs-Exo in BC chemoresistance.

#### Immune evasion

Cancer immune surveillance is a key mechanism that immune system regulates the evolution and progression of tumors. It is characterized by three essential stages: elimination, equilibrium and escape [146]. Most tumor cells are recognized and eradicated by immune system before clinical presentation. Initially, in elimination phase, innate and adaptive immune responses, such as release of proinflammatory cytokines and infiltrating immune cells lead to antitumor immunity. In second phase, pro- and antitumor immunity fail to entirely eliminate cancer cells, but keep them under control. In the last phase, tumor cells absolutely escape from immune surveillance [147, 148]. The studies revealed that BC cells can escape from immune system by regulation of gene expression likewise SOX4 [149]. Exo play a key role in immune regulation in BC by cargo expressed on their surface or transferred in the lumen [150]. Biswas et al. [151] have been unveiled that Exo released by tumor-educated MSCs can enhance development of BC through stimulating differentiation of MDSCs into highly immunosuppressive M2-polarized macrophages at tumor beds. Mechanistically, MSCs-Exo encapsulated in TGF-b, C1q, and semaphorins can stimulate myeloid tolerogenic activity not only through driving the overexpression of PD-L1 in immature myelomonocytic precursors and committed CD206 + macrophage but also through prompting differentiation of MHC class II + macrophages with increased L-Arginase activity and IL-10 production at tumor beds. The results suggested that both mechanisms can increase the BC progression. In brief, MSCs-Exo act as an inhibitory mediators of immune cells in BC.

## Conclusion

In recent years, various studies have been revealed that MSCs play a vital role in tumor progression and tumor therapy. Exo are developing as important vehicles in the correlation between MSCs and cancer cells. MSCs-Exo has attracted the attention in therapeutic methods because of their relative abundance of sources and ease of isolation. In addition, other composition of Exo such as lipid and protein that increase Exo stability are other properties that make Exo as perfect transporters. Accumulating evidence has been indicated that MSCs-Exo can exert the promotive and inhibitory effects at different phases of BC development and their application as drug delivery vehicles in cancer therapy. For the first time, we reviewed the dual function of MSCs-Exo in cell proliferation, migration, metastasis, immune evasion, angiogenesis, chemotherapy, and dormancy of BC, thus suggest a more effective strategy for BC therapy. Nevertheless, the study of MSCs-Exo is facing with many questions that is needed to be addressed. For instance, MSCs-Exo from different sources may carry different contents, thereby affecting their effect on BC cells. HUC-MSCs-Exo can suppress development of BC, while AD-MSCs-Exo can induce BC progression. In addition, AD-MSCs-Exo enhanced the migration of MCF7 cells, while inhibited the proliferation of MDA-MB-231 cells, proposing that MSCs-Exo can play a contradictory role in different types of BC. Entirely, according to studies that we mentioned in this review, we can suppose that MSCs-Exo are multifaceted players of BC development and are affected through various factors.

## Data Availability

Not applicable.

## Abbreviations

AD: Adipose tissue
BC: Breast cancer
BM: Bone marrow
CAFs: Cancer-associated fibroblasts
CircRNAs: Circular RNAs
CSCs: Cancer stem cells
EMT: Epithelial-to-mesenchymal transition
EVs: Extracellular vesicles
Exo: Exosomes
ESCRT: Endosomal-sorting complex required for transport
FGF: Fibroblast growth factor
HUC: Human umbilical cord
HIF-1α: Hypoxia-inducible factor-1alpha
ILV: Intraluminal vesicle
IL-8: Interleukin-8
lncRNAs: Long non-coding RNAs
MSCs: Mesenchymal stem cells
MUC1: Metastasis gene mucin 1
MVs: Microvesicles
MVB: Multivesicular bodies
miRNAs: MicroRNAs
mRNA: Messenger RNA
MSCs-Exo: Mesenchymal stem cell-derived exosomes
MDSCs: Myeloid-derived suppressor cells
PM: Plasma membrane
mTOR: Rapamycin
TNBC: Triple-negative breast cancer
TME: Tumor microenvironment
TAMs: Tumor-associated macrophages
TRIM59: Targeting tripartite motif 59
VEGF: Vascular endothelial growth factor
